# Deep Learning to Obtain Simultaneous Image and Segmentation Outputs From a Single Input of Raw Ultrasound Channel Data

**DOI:** 10.1109/TUFFC.2020.2993779

**Published:** 2020-11-24

**Authors:** Arun Asokan Nair, Kendra N. Washington, Trac D. Tran, Austin Reiter, Muyinatu A. Lediju Bell

**Affiliations:** Department of Electrical and Computer Engineering, Johns Hopkins University, Baltimore, MD 21218 USA.; Department of Biomedical Engineering, Georgia Institute of Technology, Atlanta, GA 30332 USA; Department of Biomedical Engineering, Emory School of Medicine, Emory University, Atlanta, GA 30322 USA.; Department of Electrical and Computer Engineering, Johns Hopkins University, Baltimore, MD 21218 USA.; Department of Computer Science, Johns Hopkins University, Baltimore, MD 21218 USA.; Department of Electrical and Computer Engineering, Johns Hopkins University, Baltimore, MD 21218 USA; Department of Computer Science, Johns Hopkins University, Baltimore, MD 21218 USA; Department of Biomedical Engineering, Johns Hopkins University, Baltimore, MD 21218 USA

**Keywords:** Beamforming, deep learning, fully convolutional neural network (FCNN), image segmentation, neural network

## Abstract

Single plane wave transmissions are promising for automated imaging tasks requiring high ultrasound frame rates over an extended field of view. However, a single plane wave insonification typically produces suboptimal image quality. To address this limitation, we are exploring the use of deep neural networks (DNNs) as an alternative to delay-and-sum (DAS) beamforming. The objectives of this work are to obtain information directly from raw channel data and to simultaneously generate both a segmentation map for automated ultrasound tasks and a corresponding ultrasound B-mode image for interpretable supervision of the automation. We focus on visualizing and segmenting anechoic targets surrounded by tissue and ignoring or deemphasizing less important surrounding structures. DNNs trained with Field II simulations were tested with simulated, experimental phantom, and *in vivo* data sets that were not included during training. With unfocused input channel data (i.e., prior to the application of receive time delays), simulated, experimental phantom, and *in vivo* test data sets achieved mean ± standard deviation Dice similarity coefficients of 0.92 ± 0.13, 0.92 ± 0.03, and 0.77 ± 0.07, respectively, and generalized contrast-to-noise ratios (gCNRs) of 0.95 ± 0.08, 0.93 ± 0.08, and 0.75 ± 0.14, respectively. With subaperture beamformed channel data and a modification to the input layer of the DNN architecture to accept these data, the fidelity of image reconstruction increased (e.g., mean gCNR of multiple acquisitions of two *in vivo* breast cysts ranged 0.89–0.96), but DNN display frame rates were reduced from 395 to 287 Hz. Overall, the DNNs successfully translated feature representations learned from simulated data to phantom and *in vivo* data, which is promising for this novel approach to simultaneous ultrasound image formation and segmentation.

## I. Introduction

ULTRASOUND images are widely used in multiple diagnostic, interventional, and automated procedures that range from cancer detection [1], [2] to ultrasound-based visual servoing [3]. Despite this wide clinical utility, there are three pervasive challenges. First, the presence of speckle and acoustic clutter often complicates image interpretation [4], particularly during automated ultrasound-based tasks. Second, speckle, acoustic clutter, and other inherent ultrasound image features tend to confuse simple thresholding and filtering algorithms and require the use of more complex procedures to successfully perform automated segmentations [5]. Third, segmentation tasks are traditionally implemented after image formation [5], [6], which further increases the computational complexity of implementing segmentation algorithms to provide the desired segmentation result. These three challenges have the potential to be addressed by simultaneously outputting multiple desired information in parallel, directly from the raw ultrasound channel data, with the assistance of deep learning.

The field of deep learning has traditionally been applied to diagnostic ultrasound tasks, such as classification, segmentation, and image quality assessment [7]. Recently, there has been growing interest in applying deep neural networks (DNNs) to augment or replace the steps of the ultrasound image formation process. For example, there is a class of deep learning approaches that improve data quality obtained from a single plane wave transmission by enhancing the beamformed data [8]-[11]. Another class of ultrasound-based deep learning approaches produces high-quality images with reduced data sampling in order to increase frame rates [12]-[18]. Deep learning has also been used to replace portions of the beamforming process by learning the parameters of a model created during an intermediary beamforming step [19]-[23]. However, none of these methods provide an end-to-end transformation that learns information directly from raw channel data.

Prior work from our group [24]-[26] introduced DNNs that were trained purely with simulated data to successfully extract information directly from raw radio frequency (RF) single plane wave channel data, prior to the application of time delays or any other traditional beamforming steps. Similarly, Simpson *et al.* [27] introduced a method to learn the entire beamforming process without applying delays to the input data. This approach trains on real data rather than simulated data and uses focused transmissions rather than plane wave transmissions. With the exception of [26], no existing methods simultaneously provide ultrasound images and segmentation information directly from raw channel data.

One challenge with learning information directly from raw channel data is the absence of receive focusing delays. Instead, the DNN input has dimensions of time versus channels, and the DNN output has dimensions of depth versus width. Thus, the network architecture must account for the mapping of time (recorded on each channel) to depth, as well as the mapping of multiple channels (which includes temporal recordings) to a single pixel in the image width dimension, and the proposed task is, therefore, not a simple image-to-image transformation. This challenge is not present in other ultrasound-based deep learning approaches that learn image-to-image transformations using input and output data that are both represented in the same spatial domain. In addition, our previous work did not take advantage of the lower spatial frequencies available when performing this transformation with raw, complex, baseband, in-phase and quadrature (IQ) data (when compared with the higher spatial frequencies of raw RF ultrasound channel data).

**Fig. 1.**
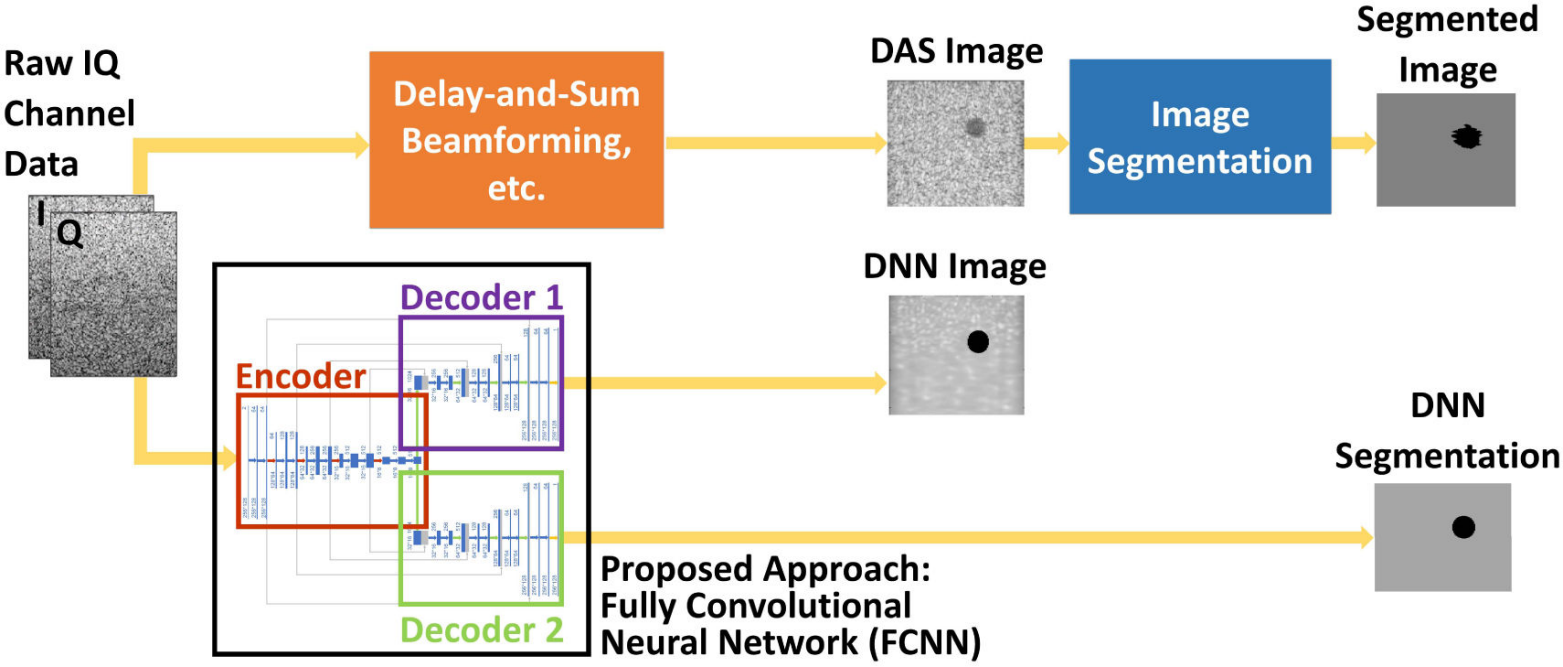
Illustration of our proposed DNN goals (bottom) in comparison to the traditional approach (top). Traditionally, raw channel data undergo DAS beamforming followed by envelope detection, log compression, and filtering to produce an interpretable DAS beamformed image, which is then passed to a segmentation algorithm to isolate a desired segment of the image. We propose to replace this sequential process with an FCNN architecture, consisting of a single encoder and two decoders, which simultaneously outputs both a DNN image and a DNN segmentation directly from raw ultrasound channel data received after a single plane wave insonification. The input is in-phase/quadrature (IQ) ultrasound data, presented as a 3-D tensor.

The primary contribution of this article is a detailed description and analysis of a DNN framework [28] that is, to the author’s knowledge, the first to replace beamforming followed by segmentation (as shown in the top of Fig. 1) with parallel B-mode and segmentation results offered as a paired network output from a single network input of raw IQ data (as shown in the bottom of Fig. 1). This parallel information may be extracted directly from the recorded echoes received after a single plane wave insonification, either before or after the application of time delays (which can be implemented in hardware), or after receiving channel data from focused transmissions. We compare these three options in this article and show that a simple modification to the input layer of a DNN can be used to accommodate each of these options. These options have the potential to simultaneously benefit both robot-based computer vision tasks (which often discard many of the details in ultrasound B-mode images through postprocessing and primarily utilize the resulting target segmentation information [3], [29]) and human observers (who may require the more familiar B-mode information to override, supervise, or otherwise interpret the output of automated and image segmentation tasks). Assuming that DNNs can be optimized to be faster than current acquisition rates [30] and provide better than current image quality with single plane wave beamforming, we also provide some guidelines to focus future efforts.

To demonstrate initial proof of principle, we focus on the detection of small, round, anechoic, cyst-like targets. This focus characterizes a range of anatomical targets, including urine-filled renal calyces (which can range from 3 to 7 mm in diameter [31]), cysts in the breast (which can be as small as 2–3 mm in ultrasound images [32] with a mean size of 2.0 ± 1.8 cm [33]), and ovarian follicles (which can range from 10 to 17 mm in width [34]). We train a task-specific DNN to target these types of structures and ignore or deemphasize structures that are not anechoic (considering that this information would otherwise be ignored through image postprocessing to achieve the proposed task). One key feature of our training approach is the use of ground-truth segmentation masks to produce enhanced beamformed images in order to enhance the identification of anechoic targets during network training. In addition, network training in this article is performed in a purely supervised manner using a fully convolutional neural network (FCNN), making the network easier and faster to train when compared with the generative adversarial network (GAN) employed in our previous article [26].

The remainder of this article is organized as follows. Section II describes our network architecture, training, and evaluation methods. Section III presents our results. Section IV includes a discussion of key insights from our results, and Section V summarizes our major conclusions.

## II. Methods

### A. Problem Formulation for Unfocused Input Channel Data

Let *I*_*d*_ be a tensor that contains downsampled IQ channel data of size *d* × *w* × *q*, where *d* is the length of each downsampled IQ signal, *w* is the IQ data image width, which is set to be equivalent to the number of transducer element receive channels, and *q* has two channels, each representing the in-phase or quadrature component of the recording. Our goal is to produce one DNN beamformed image *D* and one segmentation map prediction *S*_*p*_, each with dimensions *d* × *w*, using *I*_*d*_ as input. We employ an FCNN with trainable parameters *θ* to learn the optimal mapping of *I*_*d*_ → *y* that produces acceptable images for robotic automation and human supervision, where *y* is the reference for the optimal mapping. This reference consists of a true segmentation map *S*_*t*_ and the corresponding enhanced beamformed image *E*. Thus, *y* describes the tuple (*E*, *S_t_*).

### B. Network Architecture

**Fig. 2.**
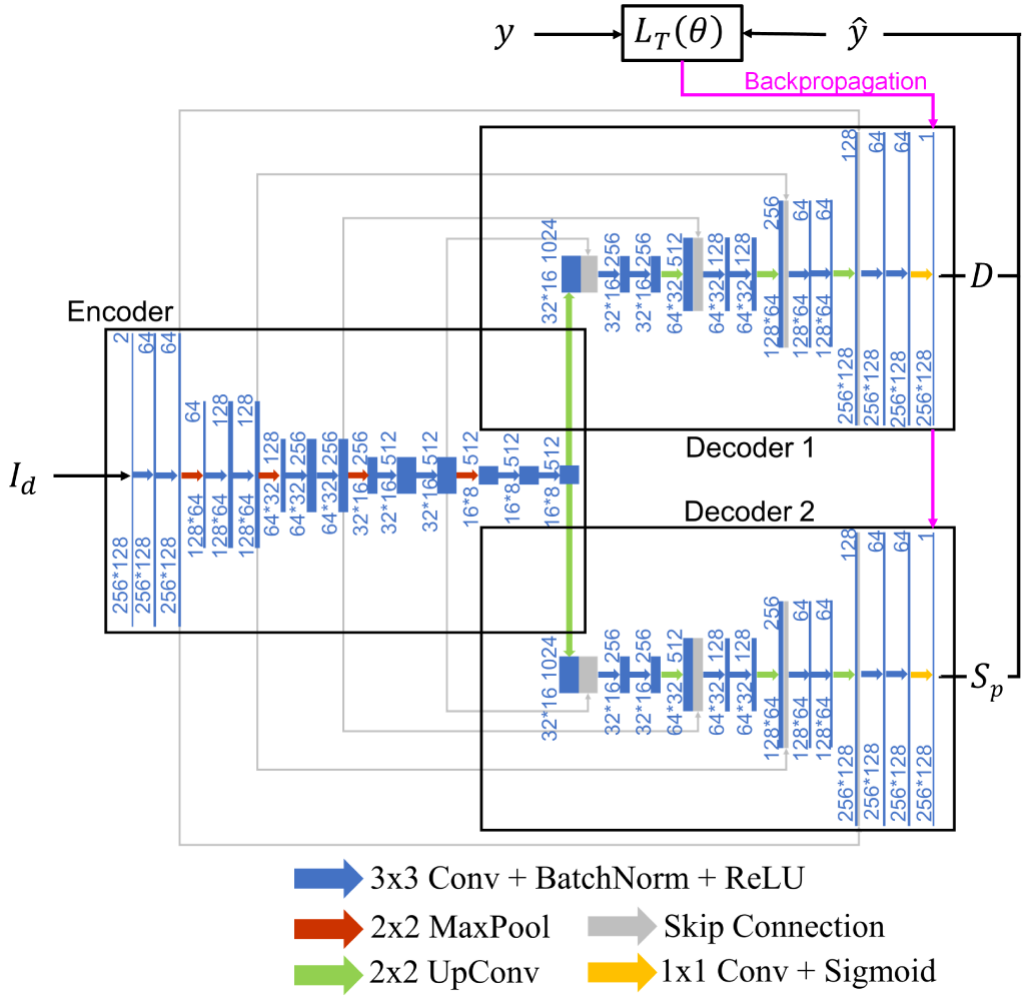
FCNN architecture and training scheme for simultaneous DNN image and DNN segmentation generation.

Our DNN architecture, shown in Fig. 2, was designed based on the U-Net [35] architecture for biomedical image segmentation, possessing a single encoder adopting the VGG-13 [36] encoder with batch normalization (BatchNorm) [37] layers to stabilize training and speed up convergence. There is one encoder, which takes the input and passes it through a series of ten 3 × 3 convolutional layers and downsamples in the spatial domain using 2 × 2 max-pooling (MaxPool) layers while simultaneously increasing the number of feature channels in the data. This process is followed by two decoders, each with nine convolutional layers. One decoder produces a DNN image, *D*(*I*_*d*_; *θ*), while the second decoder produces the DNN segmentation image, *S*_*p*_(*I*_*d*_; *θ*). The structures of the decoders are identical, each having a similar architecture to the encoder but mirrored, with 2 × 2 upconvolutional (UpConv) layers performing upsampling in the spatial domain and simultaneously decreasing the number of feature channels in the data. Both decoders have a sigmoid nonlinearity in the last layer, ensuring that the final predicted DNN image or DNN segmentation is restricted to be between 0 and 1. In addition, skip connections [38] are implemented to copy extracted features from the encoder to the decoder at the same scale (as in [35]). The skip connections enable the network to learn finer details that might otherwise be lost as a result of downsampling, enhance the flow of information through the network, and reduce training time and training data requirements [35], [36].

### C. Mapping and Scaling of Network Input and Training Data

In order to consider the time-to-depth mapping described in Section I, each recorded channel data image, *I*, was downsampled from a grid size of approximately 8300 pixels × 128 pixels (time samples × receive channel number) to a grid size of 256 pixels × 128 pixels (depth × width) with linear interpolation, satisfying Nyquist criteria and resulting in *I_d_*. To achieve *I*_*d*_, each axial line in *I* (i.e., the recorded echo samples) was mapped to a fixed position in space using an input speed of sound value that is either known (for simulated data) or assumed (for experimental data). In general, the reduction of the input data size (e.g., from *I* to *I_d_*) was necessary to maintain the entire input and the corresponding output images, as well as the corresponding gradient information of the DNN, within the GPU memory during training, and to increase training and inference speed. *I_d_* was then normalized by the maximum absolute value to ensure *I*_*d*_ ∈ [−1, 1], resulting in the network input.

To scale the training data used for obtaining the DNN image output, the recorded channel data image *I* was demodulated to baseband, beamformed, downsampled, filtered to create envelope-detected data, and then log-compressed to achieve *I*_*dB*_. The demodulation, beamforming, downsampling, and filtering steps were implemented with the Ultrasound Toolbox [39]. *I*_*dB*_ was initially displayed on a log scale with a dynamic range of 60 dB (which is a common dynamic range when displaying ultrasound images). *I*_*dB*_ was then rescaled to *I*_*n*_ as follows:

(1) $$I_{n}=\frac{I_{dB}+60}{60}$$

in order to ensure *I*_*n*_ ∈ [0, 1]. This normalization is an important step for stable DNN training, as neural networks are highly sensitive to data scaling [37], and optimal performance is typically achieved when the ranges of the inputs and outputs of the network are normalized.

**Fig. 3.**
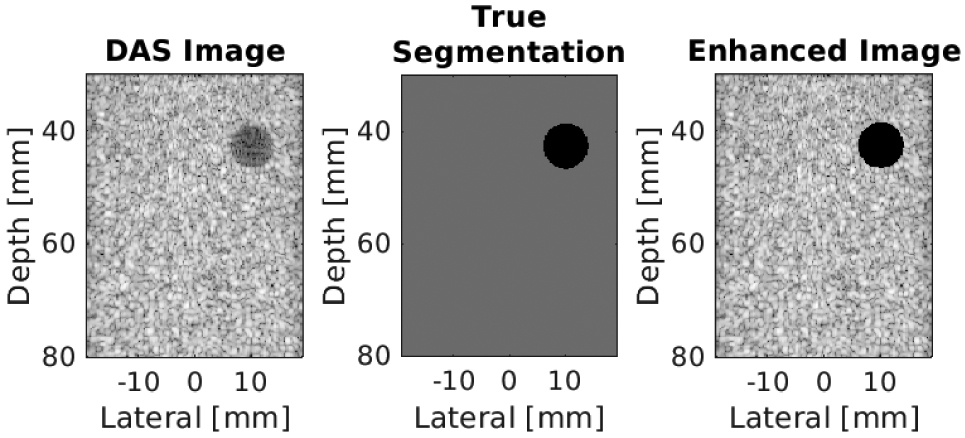
From left to right, this example shows a simulated DAS beamformed ultrasound image *I*_*n*_, the ground-truth segmentation of the cyst from surrounding tissue *S_t_*, and the corresponding enhanced beamformed image, *E* (used during network training only).

A final enhancement was applied to *I_n_* to obtain an enhanced B-mode image *E* in efforts to overcome the poor contrast and acoustic clutter limitations of single plane wave transmissions. For example, Fig. 3 shows a DAS beamformed image obtained after a single plane wave insonification of an anechoic cyst simulated with Field II [40], [41], followed by the true segmentation and the enhanced image used during network training only. The rationale for this enhancement is that the cyst is intrinsically anechoic, but the visualized cyst in the DAS beamformed image contains acoustic clutter (e.g., the sidelobe responses of the scatterers in the surrounding tissue region extending into the anechoic cyst region). Our goal is to ideally obtain better quality images than that of DAS images (and not to simply replicate poor DAS image quality during training). Toward this end, the pixel labels obtained from the input echogenicity map (which is also considered as the true segmentation mask *S*_*t*_) were used to set the pixel values of the anechoic regions in *I_n_* to zero while preserving the pixel values of the surrounding tissue, with the intention of removing the clutter observed within the cyst, thereby restoring the desired anechoic appearance of the cyst, as shown in Fig. 3. Enhanced beamformed DAS images *E* were only used to train the DNN to learn the mapping function required for the estimation of the optimal network parameters *θ* by minimizing the loss between the reconstructed images $\hat{y}$ and the reference *y*, where $\hat{y}$ describes the tuple (*D*, *S*_*p*_). Note that the procedure described to obtain the enhanced images was not applied to alter any of the DNN output images.

### D. Network Training

During training, the total network loss *L*_*T*_(*θ*) was composed of the weighted sum of two losses. The first loss was the mean absolute error, or L1Loss, between the predicted DNN image *D* and the reference enhanced beamformed image *E* defined as

(2) $$\text{L1Loss}(\theta )=\frac{1}{n}\sum_{i=1}^{n}\frac{\|D_{i}(I_{d};\theta )-E_{i}{\|}_{1}}{N}$$

where ∥·∥_1_ is the *ℓ*_1_ norm, *D_i_* and *E*_*i*_ are the vectorized images for each training example, *N* is the total number of image pixels, and *n* is the total number of training examples in each minibatch (i.e., the minibatch size). The second loss was the Dice similarity coefficient, or DSCLoss, between the predicted DNN segmentation *S*_*p*_ and the true segmentation *S*_*t*_ defined as

(3) $$\text{DSCLoss}(\theta )=\frac{1}{n}\sum_{i=1}^{n}1-2\frac{|S_{p,i}(I_{d};\theta )\cap S_{t,i}|}{|S_{p,i}(I_{d};\theta )|+|S_{t,i}|}$$

where *S*_*p,i*_ and *S*_*t,i*_ are the vectorized segmentation masks for each training example. While the target segmentation mask is binary valued, the predicted segmentation mask is allowed to be continuous valued between 0 and 1 (with the range restricted by the sigmoid nonlinearity in the final layer). A pixel value of 0 in the predicted segmentation can be interpreted as the pixel being predicted as tissue with 100% confidence, and a value of 1 can be interpreted as the pixel being predicted as cyst with 100% confidence. Thus, the DSCLoss function is implemented as a soft loss, ensuring that gradient information can flow backward through the network. The total network loss was the weighted sum of the two losses defined in 2 and 3, each loss receiving a weight of one, as defined by

(4) $$L_{T}(\theta )=\text{L1Loss}(\theta )+\text{DSCLoss}(\theta )\;\;=\frac{1}{n}\sum_{i=1}^{n}\frac{\|D_{i}(I_{d};\theta )-E_{i}{\|}_{1}}{N}+1-2\frac{|S_{p,i}(I_{d};\theta )\cap S_{t,i}|}{|S_{p,i}(I_{d};\theta )|+|S_{t,i}|}.$$

In summary, the network was trained to learn $\hat{y}$, which was composed of representations of *E* and *S*_*t*_ from input *I*_*d*_, to jointly produce both the DNN image *D* and the DNN segmentation *S*_*p*_.

Unless otherwise stated, the DNN was trained using the following baseline settings. The Adam [42] optimizer used a learning rate of 10^−5^ for 25 epochs, where one epoch is defined as one pass over the entire training data set (i.e., the entire training data set is once presented to the network for training). The minibatch size for the training data set was 16.

Training was performed on a system with an Intel Xeon E7 processor and four Tesla P40 GPUs, each equipped with 24 GB of graphics memory. To relate these computer specifications to a real-time frame rate, the training time for 25 epochs was 100 min. However, we contrast this with the inference time for our network to process 51 200 images, as reported in Section III.

### E. Comparisons to Training With Receive Delays Applied

To emphasize the challenge of deep learning from unfocused channel data, the input to the architecture shown in Fig. 2 was modified to be focused channel data and the first layer of this network was modified to accept the focused channel data. Specifically, the recorded channel data image *I* was transformed to the focused data tensor *I_f_* by applying receive time delays, resulting in a 3-D tensor with the new third dimension containing the number of focused scan lines. *I_f_* was then downsampled (using the same downsampling procedure described in Section II-C to convert *I* into *I*_*d*_), followed by the subaperture summation procedure as described in [43], resulting in *I_fds_*, which is a tensor of size *d* × *w* × *q*_*s*_, where *q*_*s*_ is twice the number of subapertures, each representing the in-phase or quadrature component of the recording. Our modified goal was to input *I_fds_* to produce *D* and *S*_*p*_, each with dimensions *d* × *w*.

To perform subaperture beamforming [43], the third dimension of *I_f_* (which contains the receive delays for each scan line) was divided into 16 subapertures (i.e., 8 elements per subaperture). The delayed data corresponding to each subaperture were summed, resulting in 16 complex-valued images, one for each of the 16 subapertures. The I and Q channels of each subaperture were then grouped together within the third dimension of the tensor to give 32 feature channels in total. Although this subaperture beamforming was performed in software in this article for ease of demonstration of the feasibility of this approach, this subaperture beamforming step can also be implemented in hardware [44], which would still result in a raw channel data input to our network (yet has the expected tradeoff of increased data transfer rates).

We employed the same FCNN described in Section II-B with the exception of a modified input layer and updated trainable parameters *θ* to learn the optimal mapping of *I_fds_* → *y*. Specifically, the first layer of the architecture shown in Fig. 2 was modified to accept 32 feature channels rather than two feature channels due to the subaperture beamforming step. This modified network was then trained as described in Section II-D, after replacing *I*_*d*_ in (2)-(4) with *I_fds_*. The same computer described in Section II-D was used for training. Training time for this modified network was 315 min. However, we contrast this with the inference time for this network to process 51 200 images, as reported in Section III.

### F. Simulated Data Sets for Training and Testing

The Field II [40], [41] ultrasound simulation package was used to generate 22 230 simulations of individual anechoic cysts surrounded by homogenous tissue. We employed simulations in our training approach for two primary reasons. First, simulations enable the generation of large, diverse data sets that are required to train robust DNNs. Second, for segmentation tasks, simulations enable the specification of ground-truth pixel labels, allowing one to avoid the expensive and time-consuming step of a human annotator to provide segmentation labels.

The simulated cyst radius (*r*), lateral and axial center positions of the cyst (*x* and *z*, respectively), and speed of sound in the medium (*c*) were varied using the range and increment sizes defined in Table I. The values of *r* were 2, 3, 4, 6, and 8 mm, which is within the range of renal calyx, breast cyst, and ovarian follicle sizes [31]-[34]. These cysts were contained within a cuboidal phantom volume located between an axial depth of 30 and 80 mm, with a lateral width of 40 mm, and an elevational thickness of 7 mm. The cysts were modeled as cylinders with the same diameter in each elevational cross section. Each simulation contained a unique speckle realization, enforced by using a different seed for the random number generator. A total of 50 000 scatterers were contained within the simulated phantom to ensure fully developed speckle.

In each simulation, a single plane wave at normal incidence was simulated to insonify the region of interest. The simulated ultrasound probe matched the parameters of the Alpinion L3-8 linear array transducer, and its center was placed at the axial, lateral, and elevation center of the phantom (i.e., 0, 0, and 0 mm, respectively). The simulated probe parameters are summarized in Table II. The one exception to matching the real hardware system was a simulated sampling frequency of 100 MHz (rather than the 40-MHz sampling frequency of the Alpinion ultrasound scanner used to acquire the experimental phantom and *in vivo* data described in Sections II-G and II-H, respectively) in order to improve the Field II simulation accuracy [40], [41].

A total of 80% of the 22 230 simulated examples were reserved for training, and the remaining 20% were used for network testing. Considering that cysts were purposely simulated to reside on the left side of the phantom (see Table I), data augmentation was implemented by flipping the simulated channel across the *x* = 0 axis to incorporate rightsided cysts in our training and testing.

To investigate the impact of depth-dependent attenuation on network training sensitivity, half of the 22 230 simulated Field II examples were simulated with an attenuation coefficient of 0.5 dB/cm-MHz, and the remaining half did not include attenuation. One DNN was trained with attenuated data, a second DNN was trained with nonattenuated data, and a third DNN was trained with the combined data set. Each network was trained for 27 625 iterations. Therefore, for this investigation, one epoch was considered to be either one pass over the combined data set (i.e., for the third DNN) or two passes over either data set with or without attenuation (i.e., for the first or second DNN, respectively), as each of these data sets is half the size of the combined data set. Using these updated definitions, the three networks were trained for 25 epochs. Unless otherwise stated (i.e., when not investigating the impact of depth-dependent attenuation), results are reported for networks trained with the combined data set.

### G. Phantom Data Sets

Channel data from a cross-sectional slice of two anechoic cylinders in a CIRS 054GS phantom located at depths of 40 and 70 mm were acquired using an Alpinion L3-8 linear array ultrasound transducer attached to an Alpinion E-Cube 12R research scanner. Two independent 80-frame sequences were acquired. The anechoic targets were consistently in the left or right half of the image for each acquisition sequence, achieved by manually flipping the ultrasound probe. In addition, the channel data corresponding to each of the 80 frames in each sequence were flipped from left to right, producing a data set consisting of 320 total images in order to test the generalizability of the trained networks. The ground truth for this phantom data set was specified by manually annotating pixels in the beamformed ultrasound image as cyst or tissue. When quantitatively evaluating these phantom examples, the mean result for the two anechoic cysts in the same image is reported, unless otherwise stated.

### H. In Vivo Data

An 80-frame sequence of *in vivo* data from a simple anechoic cyst surrounded by breast tissue (denoted as Cyst #1) was acquired using an Alpinion L3-8 linear array transducer with parameters summarized in Table II. Each plane wave acquisition was flipped from left to right to double this *in vivo* test data set size. The ground truth for this *in vivo* data set was specified by manually annotating pixels in the beamformed ultrasound image as cyst or tissue. In addition, the channel data input *I*_*d*_ was cropped to minimize the presence of bright reflectors that were not included during training. Because bright reflectors were not similarly prevalent after subaperture beamforming, the channel data input *I_fds_* was not cropped until after images were created in order to match the field of view for more direct comparisons to the results obtained with input *I*_*d*_.

To highlight the versatility of the DNN trained with *I_fds_*, this DNN was evaluated with a ten-frame sequence of an *in vivo* simple cyst surrounded by breast tissue (denoted as Cyst #2), which was originally acquired for the separate study reported in [45]. These data were acquired with focused (rather than plane wave) transmissions, using an Alpinion L8-17 linear array transducer with parameters for the acquisition listed in Table II. We include this acquisition in this article to demonstrate that plane wave input data are not a requirement for the DNN trained with focused data. The ultrasound probe also has a range of different parameters (including transmit frequency) when compared with the L3-8 linear array, which was simulated and used to train the DNN, as reported in Table II.

In addition to the channel data described earlier, clinical screenshots of the two *in vivo* cysts were additionally acquired with the Alpinion E-Cube 12R to assist with manual annotations of the cyst boundaries for ground-truth segmentations. For Cyst #1, a noticeable deformation occurred between the acquisitions due to the sequential acquisition of clinical reference images followed by plane wave data acquisitions. Therefore, the clinical B-mode image was stretched and scaled and only used to help guide the segmentation boundary definition. The acquisition of all *in vivo* data was performed after informed consent with approval from the Johns Hopkins Medicine Institutional Review Board.

### I. Comparison With Sequential Approaches

Results obtained with the trained DNNs were compared against four alternative and sequential approaches, namely DAS beamforming followed by nonlocal means (NLM), binary thresholding, NLM combined with binary thresholding, and a baseline U-Net architecture. The NLM [46], [47] and binary thresholding algorithms were implemented in MATLAB on an Intel Xeon E 5645 CPU with a clock speed of 2.40 GHz. NLM served as a baseline image smoothing algorithm. Most hyperparameters were set to their default values (i.e., the “SearchWindoSize” hyperparameter was set to 21 and the “ComparisonWindowSize” hyperparameter was set to 5), with the exception of the “DegreeOfSmoothing” hyperparameter, which was set to 0.1.

Binary thresholding followed by morphological filtering (abbreviated as BT) was implemented as described in [6], [48], [49] to compare the DNN segmentations. To summarize our BT implementation, the mean of the normalized DAS B-mode image (*I*_*n*_) was calculated, and the binarization decision threshold value was set as 0.70 times the mean pixel value. Pixels above and below the threshold were labeled as tissue and cyst, respectively. Connected components labeled as cyst tissue smaller than 50 pixels (i.e., an area of approximately 3 mm^2^) were removed to eliminate false positives. Morphological closing (i.e., a dilation followed by an erosion) was then performed with a disk element of radius 1 pixel to fill in gaps in the segmentations. Morphological dilation was then performed using a disk element of radius 2 pixels to expand the cyst segmentations (considering that previously implemented steps tend to underestimate cyst size). Hyperparameter tuning was performed to choose the baseline hyperparameters.

DAS beamforming followed by NLM and then BT (i.e., DAS + NLM + BT) was implemented to produce sequential segmentation and speckle reduced images for comparison to the parallel outputs produced by the DNN from raw IQ channel data. Finally, to compare the results with the current state of the art for ultrasound image segmentation, a baseline U-Net [35] network with a single encoder and a single decoder was implemented. This network was trained to predict a segmentation mask *S*_*p*_(*I*_*n*_; *θ*) from input *I*_*n*_ using *S*_*t*_ as the ground truth. We employed the same FCNN described in Section II-B with the exception of a modified input layer, a single decoder module, and updated trainable parameters *θ* to learn the optimal mapping of *I*_*n*_ → *S*_*p*_. Specifically, the first layer of the architecture shown in Fig. 2 was modified to accept one feature channel rather than two feature channels due to the input being the normalized DAS B-mode image *I_n_*. In addition, as only the DNN segmentation is being produced, only one decoder module is needed. This modified network was trained using the DSCLoss described by (3), after replacing *S*_*p,i*_(*I*_*d*_; *θ*) with *S*_*p,i*_(*I*_*n*_; *θ*). The same baseline settings and computer reported in Section II-D were used during training.

### J. Evaluation Metrics

1. *Dice Similarity Coefficient (DSC):* DSC quantifies overlap between two segmentation masks [50]. The DSC between the predicted DNN segmentation, denoted by *S*_*p*_ and the true segmentation, denoted by *S*_*t*_, is defined as
  (5) $$\text{DSC}(S_{p},S_{t})=2\frac{|S_{p}\cap S_{t}|}{|S_{p}|+|S_{t}|}.$$
  A perfect DNN segmentation produces a DSC of 1. Prior to display and evaluation, the predicted segmentation mask was binarized using a threshold of 0.5, considering that a predicted pixel value >0.5 indicates that the network is more confident that the pixel is cyst than tissue (and vice versa for pixel values < 0.5).
2. *Contrast:* Contrast is fundamentally a measure to quantify the differences between the minimum and maximum values in an image, particularly for regions inside and outside an anechoic cyst, respectively. This metric is defined as
  (6) $$\text{Contrast}=20\;\log_{10}\left(\frac{S_{i}}{S_{o}}\right)$$
  where *S*_*i*_ and *S*_*o*_ represent the mean of individual uncompressed signal amplitudes *s*_*i*_ and *s*_*o*_ in selected regions of interest (ROIs) inside and outside the cyst, respectively, taken from the normalized image *I*_*n*_ [see (1)]. The ROI inside the cyst was automated as a 2-mm-radius circular region centered at the cyst center for the simulated and phantom examples and a 1.5-mm-radius circular region for the more irregularly shaped *in vivo* examples. The choice to automatically use a small circular region about the cyst center was made to avoid manual ROI selection across the thousands of simulation and phantom test sets, yet still ensure that the results would be a meaningful assessment of the difference in signal amplitude inside and outside the detected cyst region. This automated ROI selection is additionally intended to prevent the inclusion of misclassifications (e.g., cyst pixels at the cyst boundary detected as tissue and vice versa), which are instead evaluated with the gCNR metric [51]. The ROI outside of the cyst was the same size as the inside ROI and was located at the same depth as the cyst. These ROIs were used to calculate the contrast of DNN, DAS beamformed, and enhanced beamformed images.
  Because the desired DNN output image was log-compressed with a chosen dynamic range of 60 dB, an uncompressed signal *s* was first calculated as
  (7) $$s=10^{s_{\text{dB}}∕20}$$
  where *s* refers to *s_i_* or *s_o_* (i.e., the subscripts were removed for simplicity) and *s*_dB_ is the log-compressed equivalent of *s*. The values of *s* were then used to calculate *S_i_* and *S_o_* in (6). Note that the maximum dynamic range of our network is 60 dB, which translates to a maximum possible contrast of 60 dB in the DAS beamformed and enhanced beamformed images.
3. *Signal-to-Noise Ratio (SNR):* Tissue SNR quantifies the smoothness of the background region surrounding the cyst, which is defined as
  (8) $$\text{SNR}=\frac{S_{o}}{\sigma_{o}}$$
  where *σ*_*o*_ represents the standard deviation of individual uncompressed signal amplitudes *s_o_* in the selected ROI outside the cyst [i.e., the same ROI used to calculate contrast in (6)]. The enhanced beamformed image contains the same tissue background as the DAS beamformed image and therefore has an identical SNR to the DAS beamformed image.
4. *Generalized Contrast-to-Noise Ratio (gCNR):* The gCNR was recently introduced as a more accurate measure of lesion detectability in comparison to CNR [51], and it calculated as
  (9) $$\text{gCNR}=1-\sum_{x=0}^{1}\underset{x}{\text{min}}\{p_{i}(x),p_{o}(x)\}$$
  where *p*_*i*_(*x*) and *p*_*o*_(*x*) are the probability mass functions of *s_i_* and *s_o_*, respectively. Considering that gCNR is intended to measure cyst detection probability, choosing the ROIs defined for contrast would bias gCNR toward better results by only providing a subset of pixels within the cyst region. Therefore, *s_i_* for the gCNR metric was updated to be the ground-truth cyst segmentation within *S*_*t*_, and *s*_*o*_ was expanded to be the same size and located at the same depth as *s_i_* .
5. *Peak SNR (PSNR):* PSNR quantifies the similarity of the generated DNN image to the reference enhanced beamformed image, considering the pixel values both inside and outside the cyst in order to provide a single value defining a global quality estimate, which is defined as
  (10) $$\text{PSNR}(D,E)=10\;\log_{10}\left(\frac{{\text{MAX}}_{E}^{2}}{\text{MSE}}\right)$$
  (11) $$=10\;\log_{10}\left(\frac{1}{\frac{\|D-E{\|}_{2}^{2}}{N}}\right)$$
  where ∥·∥_2_ is the *ℓ*_2_ norm, *D* and *E* are the vectorized DNN image and the reference enhanced beamformed image, respectively, *N* is the number of pixels in the images, and MSE is the mean square error between *D* and *E*. Because *E* ∈ [0, 1], MAX_*E*_ (i.e., the maximum absolute pixel value of image *E*) is equal to 1.
6. *Coefficient of Variation (CV):* To study the effect of minimal (e.g., due to hand tremors) to no perturbations in the phantom data across a given acquisition sequence, the CV of the contrast, SNR, and gCNR metrics was calculated as
  (12) $$\text{CV}=\frac{\sigma }{\mu }\times 100\%$$
  where *μ* is the mean metric value across multiple acquisitions and *σ* is the standard deviation of the metric across the same acquisitions. CV was calculated for both DNN and beamformed images.
7. *Processing Times:* Processing times for DAS beamforming, DNN performance, and NLM, BT, and U-Net comparisons were calculated. The processing time to perform DAS beamforming with a single plane wave was approximated from the GPU beamformer processing times for 25 plane waves reported in [52]. We included the times to perform the DAS operations (i.e., FocusSynAp and ChannelSum, respectively) and divided the summation of the reported processing times for these operations by 25 to achieve a processing time estimate for a single plane wave. The reported processing times were implemented on an NVIDIA Titan V GPU.

The processing times for NLM and BT were calculated after applying these algorithms to the entire test set of 4554 simulated B-mode images. The total processing time was then divided by the total number of images processed to provide an estimate of the time to produce a single image. This time was added to the time per image reported for DAS beamforming to estimate the times for DAS+NLM, DAS+BT, and DAS+NLM+BT.

To calculate the processing times for U-Net segmentation, a mini batch of 512 tensors of simulated *I*_*n*_ were input 100 times into the trained network, and the total processing time was divided by the total number of images processed (i.e., 51 200 images). This time was added to the time per image reported for DAS beamforming to estimate the times for DAS+U-Net.

To calculate the processing time per image during DNN testing, a mini batch of 512 tensors of simulated *I*_*d*_ or *I_fds_* were input 100 times into the DNN trained with unfocused or focused data, respectively. The total processing time for each DNN was then divided by the total number of images processed (i.e., 51 200 images) to provide an estimate of the time that it would take to process a single image for each DNN.

Calculated processing times were then inverted to provide the expected frame display rates. Although these reports combine the CPU and GPU performances, we only perform the direct comparisons of CPU-to-CPU and GPU-to-GPU processing times implemented on the same computer.

### K. Exclusion Criteria

As demonstrated in our previous work [25], higher DSCs are achieved with larger cysts compared with smaller cysts. In addition, small cysts have a greater potential to be missed, which is quantified as a DSC of approximately zero. Based on this knowledge, we prioritize a fair comparison of the multiple network parameters, which we define as a minimum DSC ≥0.05. This criterion was required for the network trained with the baseline settings reported in Section II-D, and test cases that did not meet this basic criterion with this baseline test set were excluded from the results reported in this article. Note that our exclusion criteria were only applied to one of several test sets, and the excluded images from this test set analysis were then excluded in the subsequent test sets (i.e., the exclusion criteria were not repeated for each test set).

The resulting detection rate is listed for each cyst radius in Table III. Overall, no experimental phantom or *in vivo* data met our exclusion criteria, and the network successfully detected the simulated cysts in 4274 out of 4554 test examples. Table III also indicates that segmentation failure primarily occurs with 2-mm-radius cysts. The remaining cyst examples were successfully detected, and we prefer to limit our methodology feasibility assessments to these cases. Therefore, the results in Section III-A are reported for this subset of the simulated test set. This information can additionally be used to avoid applications of our approach to cysts smaller than 2 mm radii, which are challenging for the DNN to detect, likely due to the presence of acoustic clutter in the single plane wave image.

## III. Results

### A. Simulation Results

**Fig. 4.**
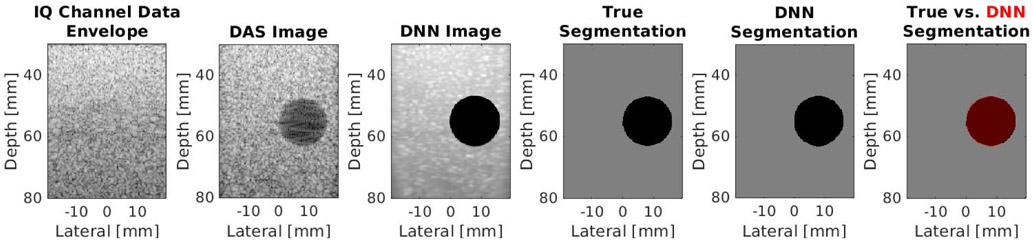
Simulation result showing, from left to right, raw IQ channel data (displayed with 60-dB dynamic range after applying envelope detection and log compression), a DAS beamformed ultrasound image, a DNN image produced by our network, the known segmentation of the cyst from surrounding tissue, the DNN segmentation predicted by our network, and an image with a red transparent overlay of the DNN segmentation over the true segmentation.

Fig. 4 shows an example simulated test case from the DNN architecture shown in Fig. 2 using the baseline settings noted in Section II-D. From left to right, this example shows the simulated raw IQ channel data, the corresponding DAS beamformed ultrasound and DNN image, the known segmentation of the cyst from surrounding tissue, the DNN segmentation predicted by our network, and the DNN segmentation overlaid on the true segmentation. This example produces a DSC of 0.98, a contrast of −42.11 dB, an SNR of 3.06, a gCNR of 0.99, and a PSNR of 20.32 dB. The test set (excluding the cases noted in Section II-K) produced mean ± one standard deviation DSC of 0.92 ± 0.13, contrast of −40.07 ± 11.06 dB, SNR of 4.29 ± 1.26, gCNR of 0.95 ± 0.08, and PSNR of 20.19 ± 0.40 dB.

**Fig. 5.**
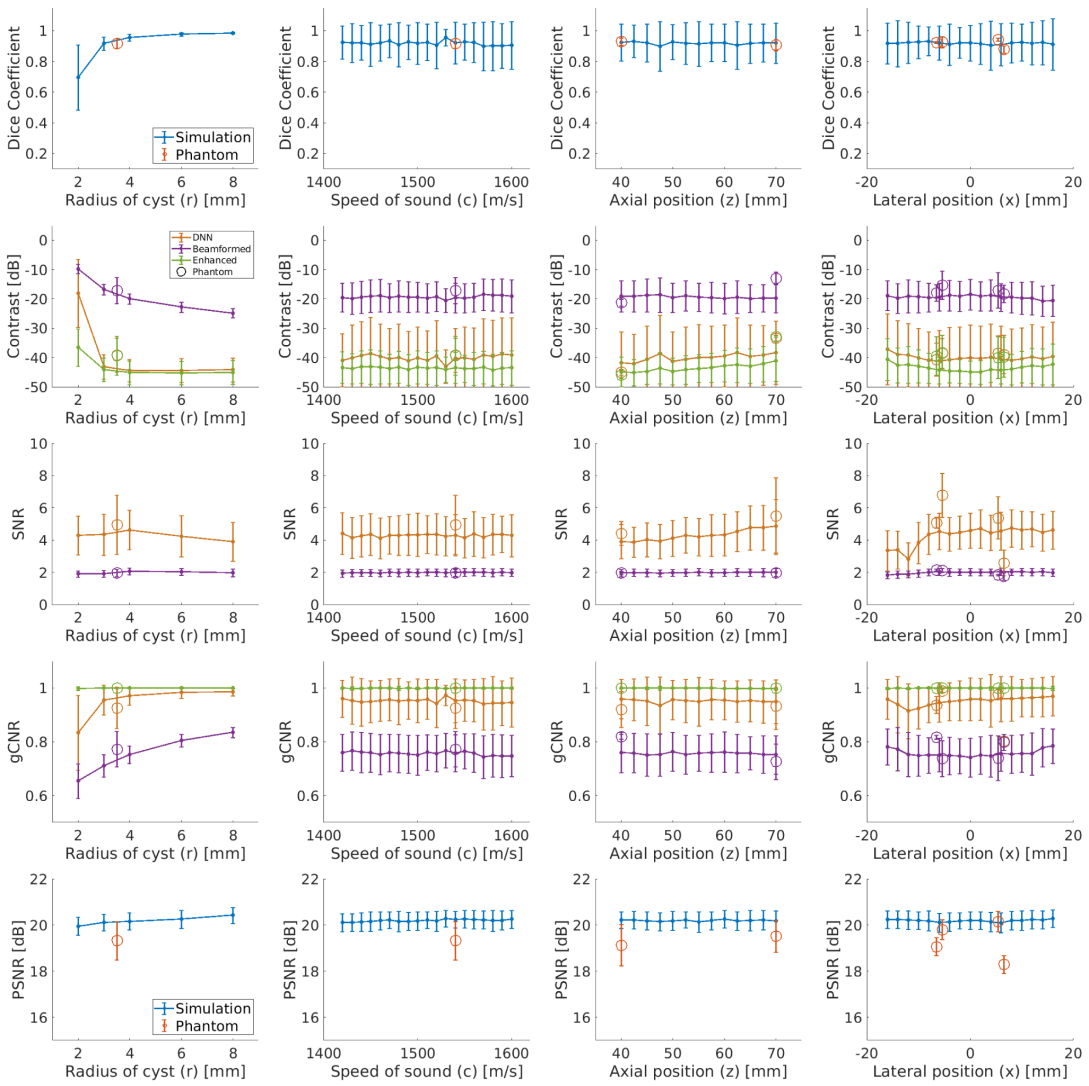
Aggregated mean (from top to bottom) DSC, contrast, SNR, gCNR, and PSNR ± one standard deviation as a function of (from left to right) variations in *r, c, z,* and *x* for simulated and experimental phantom results. Experimental phantom results are displayed using unfilled circle markers. “Enhanced” indicates the performance of the enhanced B-mode images that were used for DNN training, as described in Section II-C, and they represent the limits to an ideal DNN performance.

Fig. 5 shows the aggregated mean DSC, contrast, SNR, gCNR, and PSNR ± one standard deviation as a function of (from left to right) variations in *r*, *c*, *z*, and *x* for simulated results and phantom results. The simulation results in Fig. 5 reveal that the smaller, 2-mm radii cysts yield the worst DNN segmentations with a mean DSC of 0.70. The DSC rises to 0.99 for 8 mm cysts. Similarly, as *r* increases, contrast improves from −18.12 to −44.20 dB, gCNR improves from 0.83 to 0.97, and PSNR improves from 19.95 to 20.42 dB. Unlike DSC, contrast, gCNR, and PSNR, SNR does not change as *r* increases. The DSC, contrast, SNR, and gCNR results are otherwise relatively constant as functions of the remaining parameters (i.e., *c*, *z*, and *x*).

Focusing on the contrast results in Fig. 5, the contrast of the DNN images approaches that of the enhanced beamformed image as *r* increases and is consistently superior to the contrast of the traditional DAS beamformed images, with a mean contrast improvement measuring 20.71 dB. In addition, Fig. 4 shows that the tissue texture is smoother in the DNN images when compared with the DAS beamformed images. The quantitative SNR results in Fig. 5 support this observation, and the mean SNR improvement is 2.30. These two improvements combine to produce a mean gCNR improvement of 0.19 when DNN images are compared with DAS beamformed images.

### B. Phantom Results

**Fig. 6.**
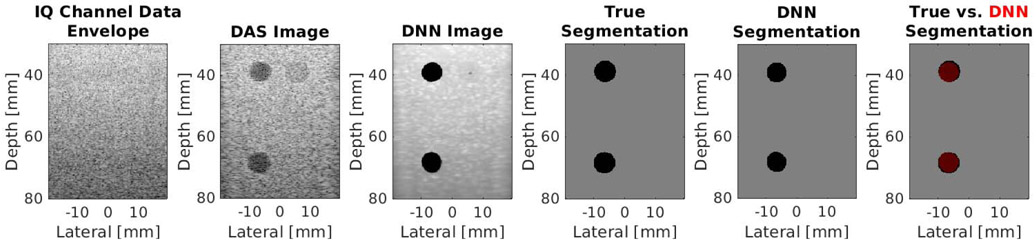
Experimental phantom result showing, from left to right, raw IQ channel data (displayed with 60-dB dynamic range after applying envelope detection and log compression), a DAS beamformed ultrasound image, a DNN image produced by our network, the known segmentation of the cyst from surrounding tissue, the DNN segmentation predicted by our network, and an image with a red transparent overlay of the DNN segmentation over the true segmentation.

Fig. 6 shows an example test case from the phantom data set. From left to right, this example shows raw phantom IQ channel data, a DAS beamformed ultrasound image and corresponding DNN image, the known segmentation of the cyst from surrounding tissue, the DNN segmentation predicted by our network, and the DNN segmentation overlaid on the true segmentation. This example produces a DSC of 0.92, a contrast of −40.69 dB, an SNR of 4.96, a gCNR of 0.93, and a PSNR of 18.97 dB. The entire test set produced mean ± one standard deviation DSC of 0.92 ± 0.03, contrast of −39.13 ± 5.86 dB, SNR of 4.96 ± 1.84, gCNR of 0.93 ± 0.08, and PSNR of 19.33 ± 0.83 dB.

The aggregated results of this entire data set as functions of *r*, *c*, *z*, and *x* are shown in Fig. 5 as unfilled circles overlaid on the previously discussed simulation results. The color of each circle corresponds to the color-coded data type listed in the legend. Fig. 5 shows that the mean DSC, contrast, and gCNR measurements for the phantom results are generally within the range of the standard deviations of these same measurements for the simulation results. However, the SNR and PSNR of the phantom results are outliers when compared with those of the simulation results because of the differences in tissue texture achieved with the DNN image.

Note that the phantom test data set consists of 160 total plane wave insonifications. Half of these acquisitions contain the two anechoic cysts on the left side of the image, and the other half (acquired with the probe physically flipped) contain the same anechoic cysts on the right side of each image. The raw data from each acquisition were then flipped, yielding a data set with a total of 320 plane waves and a total of eight individual “cyst templates.” CV was calculated for each individual cyst template, and the mean of these eight CVs was 0.12%, 2.38%, and 0.36% for DNN image contrast, SNR, and gCNR measurements, respectively. These results are comparable with those of the DAS beamformed images (i.e., contrast, SNR, and gCNR CVs of 1.19%, 0.63%, and 0.82%, respectively). This result indicates that there were minimal variations in the acquired phantom results, which were purposely acquired with minimal to no perturbations to the acquisition setup. The implication of this result is discussed in more detail in Section IV.

### C. Incorporating Attenuation

**Fig. 7.**
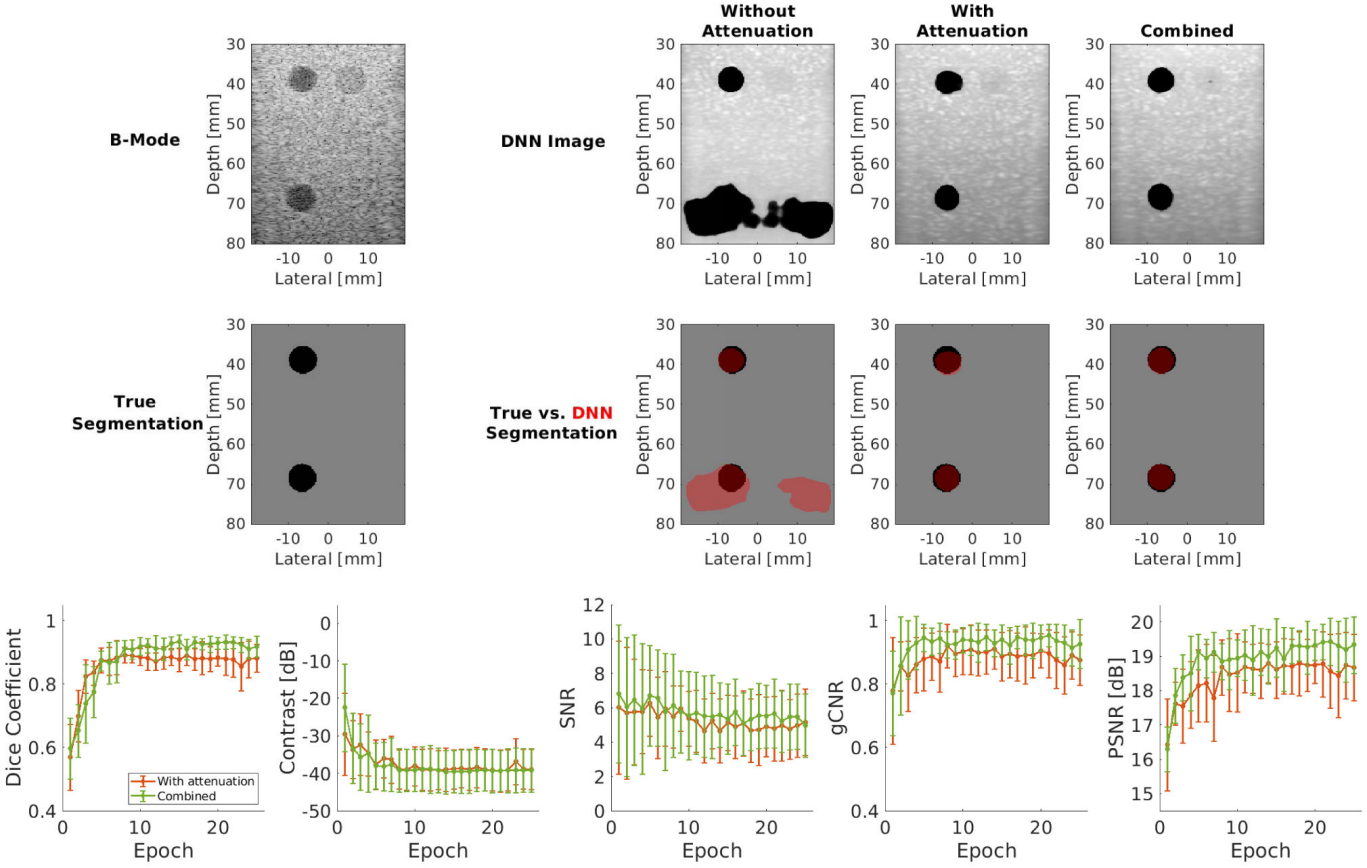
Top: attenuation results showing, from left to right, the DAS beamformed image and ground-truth segmentation reference pair, the corresponding outputs of the network trained with nonattenuated data, attenuated data, and the combined data set of both attenuated and nonattenuated data. Bottom: aggregated attenuation results, showing mean DSC, contrast, SNR, gCNR, and PSNR ± one standard deviation as a function of epoch.

Fig. 7 (top) shows example test cases from the three networks trained with, without, and both with and without attenuation combined. From left to right, the first column of images in Fig. 7 displays the DAS beamformed image along with the true segmentation, the second column displays the output of the network trained without attenuation, the third column displays the output of the network trained with attenuated data, and the fourth column displays the output of the network trained with the combined data set of both attenuated and nonattenuated data. The example output from the network trained with nonattenuated data produced DSC, contrast, SNR, gCNR, and PSNR of 0.66, −41.64 dB, 3.08, 0.64, and 14.16 dB, respectively. The network trained with attenuated data produced DSC, contrast, SNR, gCNR, and PSNR of 0.86, −40.27 dB, 4.79, 0.85, and 18.16 dB, respectively, representing improved DSC, SNR, gCNR, and PSNR with similar contrast. Additional improvements were achieved when training with both attenuated and nonattenuated data, producing DSC, contrast, SNR, gCNR, and PSNR of of 0.92, −40.69 dB, 4.96, 0.93, and 18.97 dB, respectively.

Fig. 7 (bottom) shows the aggregated mean DSC, contrast, SNR, gCNR, and PSNR ± one standard deviation as a function of the number of epochs for the networks trained with attenuated data and with the combined data set of both attenuated and nonattenuated data. When trained with the combined data set, it is remarkable that the addition of nonattenuated data does not significantly impact the performance of the network in spite of the test phantom data set having tissue attenuation. Instead, the inclusion of nonattenuated data seems to be responsible for a subtle boost in performance. For example, when the measured DSC is averaged over epochs 11–25, this average improves from 0.88 when the network is trained with the attenuated data set to 0.92 when the network is trained with the combined data set. Similarly, when each metric result is averaged over all epochs, SNR improves from 5.26 to 5.73, gCNR improves from 0.88 to 0.92, and PSNR improves from 18.38 dB to 18.98 dB. Contrast results are similar between the two networks.

### D. Comparisons Between Focused and Unfocused Input Data

**Fig. 8.**
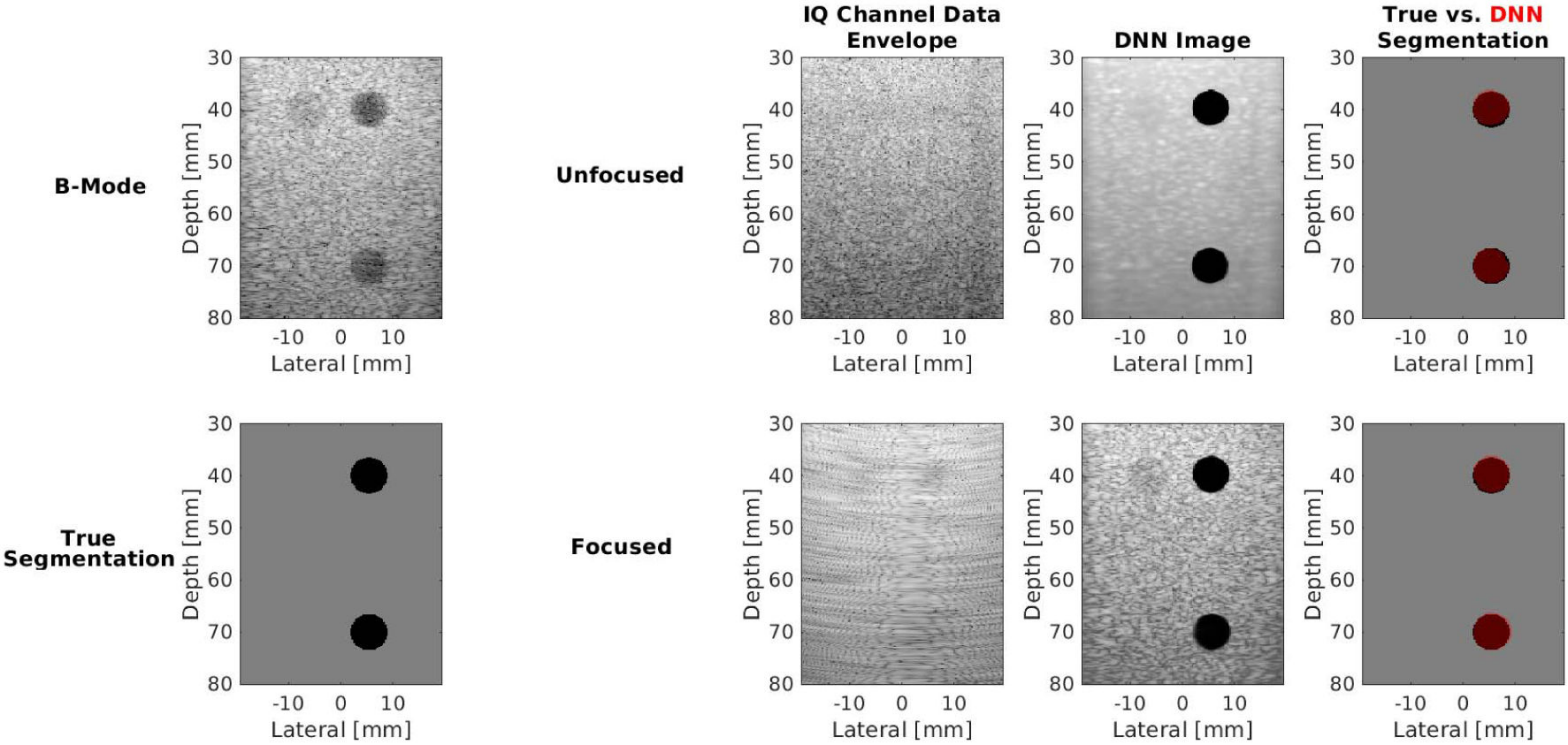
Comparison of *I*_*d*_ and *I*_*fds*_ input experimental phantom data showing, from left to right, the DAS beamformed image and ground-truth segmentation reference pair, the unfocused and focused IQ channel data envelopes of the input data *I*_*d*_ and *I*_*fds*_, respectively, and the corresponding outputs of the two DNNs. For the focused IQ channel data envelope image, a subaperture input near the center of the probe is displayed as a representation of the input to one channel of the DNN.

Fig. 8 shows the phantom images comparing unfocused input data *I*_*d*_ to focused input data *I_fds_*. The contrast, SNR, and gCNR of the image created with the focused input are −36.22 dB, 1.63, and 0.94, respectively. The corresponding values for the image created with unfocused data are −38.41 dB, 5.61, and 0.98, respectively. Therefore, these metrics are improved with unfocused data in this particular example. However, the PSNR and DSC are 20.14 dB and 0.94, respectively, with the unfocused input, compared with 22.63 dB and 0.94, respectively, with the focused input. While the higher PSNR with the focused input is due to tissue SNR that more closely resembles that of the DAS B-mode images, the similar DSC results demonstrate that the similar segmentation performance can be achieved with DNNs regardless of the inclusion of focusing. Table IV summarizes these metrics for the acquired phantom images and also compares the time required to create each DNN image.

Table IV additionally demonstrates that similar speckle SNR to the reference B-mode image is achieved when the input data are focused to include receive time delays. However, this focusing approach requires an updated network input layer with 30 additional input channels (to accept the increased input data size), as well as the additional step of subaperture beamforming, which both reduce the overall frame rates. Note that the additional step associated with subaperture beamforming is not included in the processing time results reported in Table IV, as subaperture beamforming could be implemented in hardware.

**Fig. 9.**
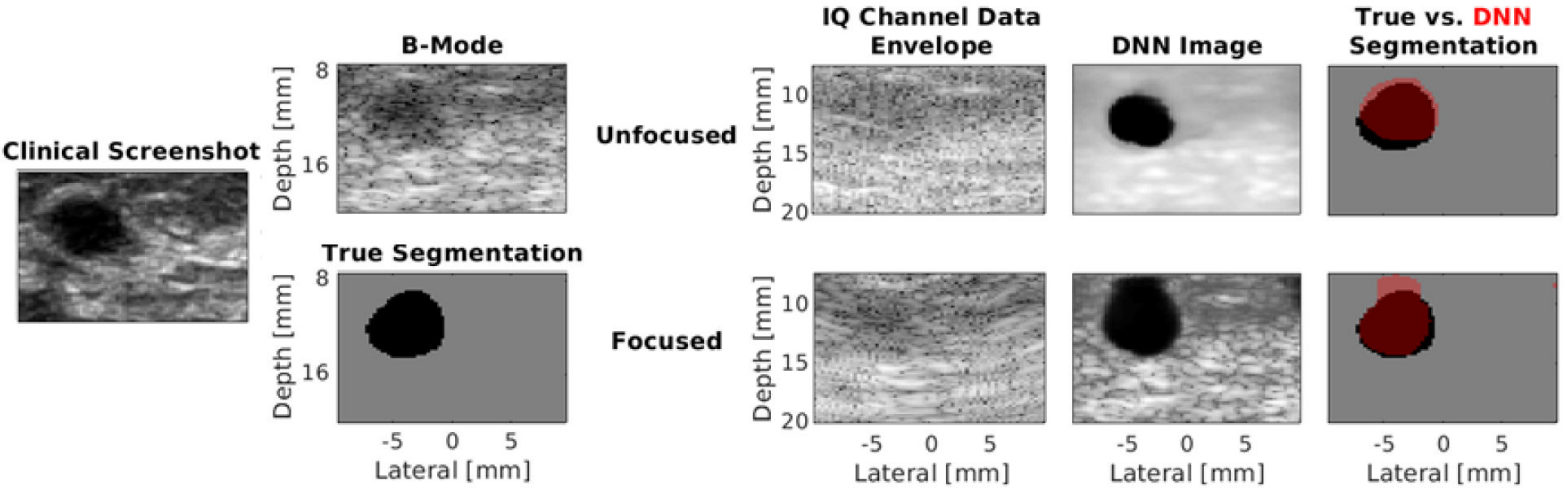
Comparison of *I*_*d*_ and *I*_*fds*_ input *in vivo* data from Cyst #1 showing, from left to right, the clinical image obtained from the scanner with an 8-MHz transmit frequency focused at a depth of 20 mm, the DAS beamformed image of Cyst #1 obtained using a single 0° incidence plane wave transmitted at 4 MHz and the corresponding ground-truth segmentation reference pair, the unfocused and focused IQ channel data envelopes (with the latter showing the envelope of a single subaperture) of the input data *I*_*d*_ and *I_fds_*, respectively, and the corresponding outputs of the two DNNs.

Fig. 9 shows the *in vivo* images of Cyst #1 comparing unfocused input data *I*_*d*_ to focused input data *I_fds_*. The DSC, contrast, SNR, gCNR, and PSNR of the outputs created with the unfocused input are 0.83, −34.89 dB, 4.57, 0.90, and 15.85 dB, respectively. Although the DSC and gCNR results are lower than the majority of examples previously shown, it is important to note that the size of Cyst #1 is approximately 3 mm in radius, and the DSC and gCNR results of this cyst are within the range of the means ± one standard deviation obtained for the 2–4-mm radii results reported in Fig. 5 (i.e., 0.70 ± 0.21 to 0.96 ± 0.2 and 0.83 ± 0.14 to 0.97 ± 0.03, respectively). In addition, SNR starts at a lower value than the phantom and simulated DAS results reported in Fig. 5; therefore, the final value obtained with the DNN is also lower than those shown in Fig. 5. Nonetheless, SNR and contrast are still improved when this DNN image is compared to the corresponding DAS B-mode image.

The DSC, contrast, SNR, gCNR, and PSNR of the outputs created with the focused input *I_fds_*, are 0.85, −21.89 dB, 0.93, 0.85, and 19.01 dB, respectively, for the example shown in Fig. 9. The DNN overestimates the proximal cyst boundary in this example, likely due to large amplitude differences at that boundary, which were not included during training. The mean ± standard deviation of the evaluation metrics for the entire 160 frames in the test data set for Cyst #1 are reported in Table IV.

Figs. 8 and 9 show that more similar speckle SNR results were obtained with phantom and *in vivo* data when *I_fds_* was the input, as summarized in Table IV. In particular, with *I_fds_* as the input, the SNRs of the phantom and *in vivo* data more closely match the SNR results reported for the corresponding DAS B-mode images. The higher tissue SNR of DNN images obtained with *I*_*d*_ as the input, when compared with corresponding DAS images, occurs because of the smoother tissue texture in these DNN images, despite both DNNs being trained with data that fundamentally contain speckle, which is caused by constructive and destructive interference from subresolution scatterers [53], [54].

These SNR results demonstrate that the DNN with *I_d_* as input is unable to learn the finer details associated with the transformation from unfocused tissue texture to traditional B-mode image speckle (which is included in the transformation *I*_*d*_ → *D*), and therefore, $\hat{y}$ is not a faithful representation of *y* from this perspective. In contrast, considering that the same network architecture was implemented after receive focusing delays were applied to the input data (and after the input layer was modified to accept this larger input data), the transformation *I_fds_* → *D* appears to be a simpler task for this DNN, which can be explained by the transformation from focused tissue texture to speckle being a more direct image-to-image transformation (particularly after the downsampling step described in Section II-D).

While the smoothing and higher SNRs observed in the output DNN images created from the unfocused input data *I_d_* may be viewed as a failure of the network from the perspective of faithful image reconstruction, from the perspective of the proposed task and the DNN goals, the higher tissue SNR and smoother tissue texture are viewed as a benefit. These achievements are aligned with the goals of maximizing achievable frame rates, deemphasizing unimportant structures, and emphasizing structures of interest for the proposed task.

**Fig. 10.**
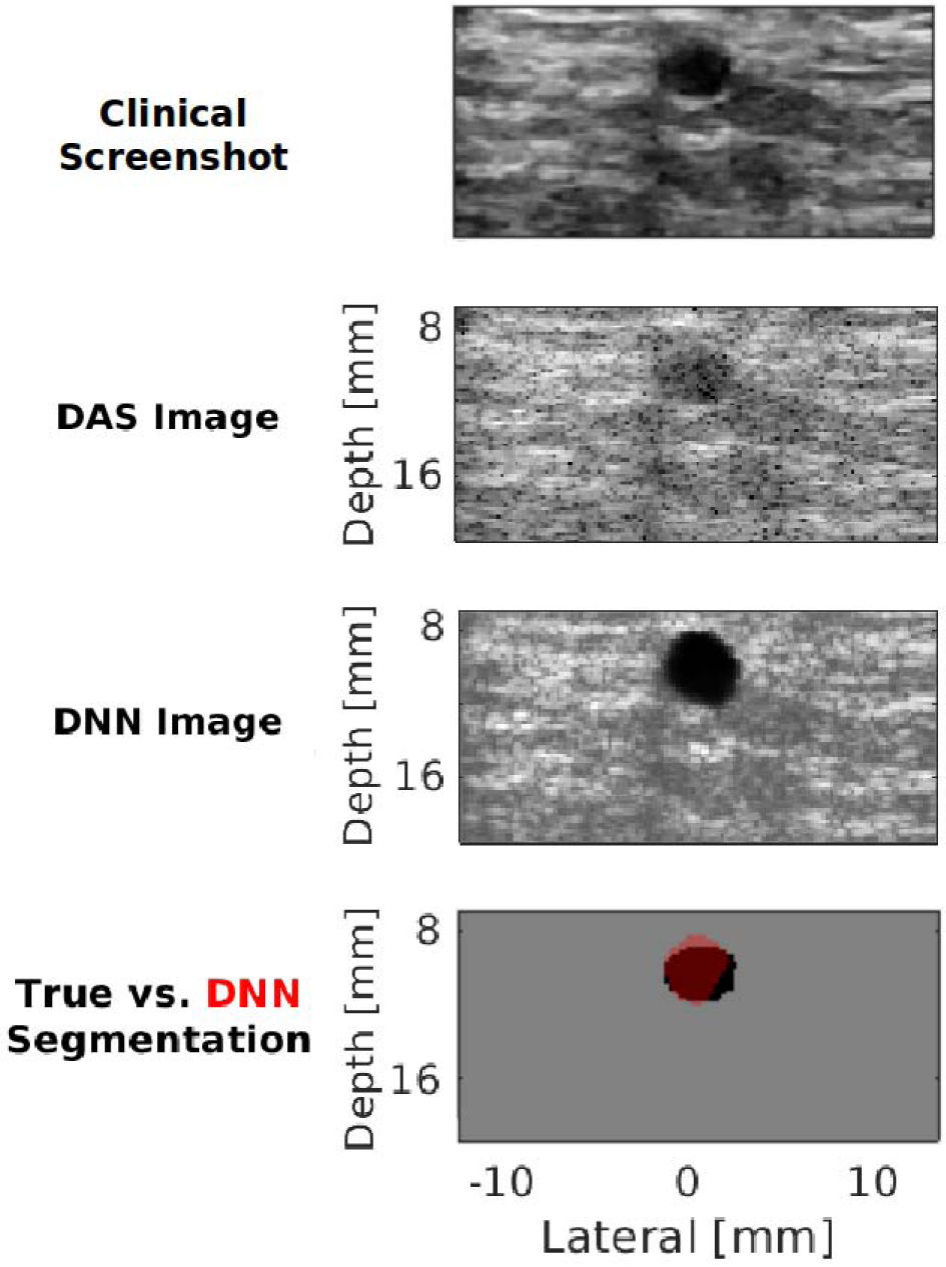
*In vivo* clinical image of Cyst #2 obtained from the scanner with a 12-MHz transmit frequency focused at a depth of 10 mm, DAS beamformed image of Cyst #2, the corresponding DNN image, and the corresponding DNN segmentation overlaid on the true segmentation.

Fig. 10 shows an additional example of this expected tradeoff between preserving fidelity and achieving task-specific image reconstruction goals with Cyst #2. This example was obtained from focused transmissions and with a higher transmit frequency than that used during training, thus highlighting the versatility of the DNN with *I_fds_* as input. This network produces DNN images that have a closer match to the DAS beamformed image, with remarkably higher contrast than that otherwise achieved with a single plane wave transmission. The contrast of this DNN image is qualitatively similar to that obtained with the clinical screenshot (which was acquired with focused ultrasound transmit beams). However, this DNN image contains tissue structure and speckle that can potentially confuse an observer who is not skilled with reading ultrasound images (in addition to requiring more time to produce this image in comparison to the image that would be produced with an unfocused data input). The DSC, contrast, SNR, gCNR, and PSNR for this result are 0.82, −34.18 dB, 1.50, 0.97, and 19.45 dB, respectively. The mean ± standard deviation of these metrics for the entire 20 frames in the test data set for Cyst #2 are reported in Table IV.

When comparing the presented DNN performance to more standard methods, Table IV demonstrates that although B-mode alone produces the fastest frame rates (i.e., 4000 Hz on a GPU), frame rates are expected to be reduced after image formation followed by either speckle reduction (i.e., DAS + NLM results in 75 Hz on GPU + CPU), segmentation (i.e., DAS + BT results in 455 Hz on GPU + CPU), or both speckle reduction and segmentation (i.e., DAS + NLM + BT results in 64 Hz on GPU + CPU). The DNN that accepts unfocused data has faster frame rates (i.e., 395 Hz) when compared with the DNN that accepts focused data (i.e., 287 Hz). Although implementation on two different GPU configurations confounds direct processing time comparisons, the sequential DAS + U-Net approach was faster than the parallel DNN approaches. There is also room for improvement of the parallel DNN approaches to achieve even faster frame rates than currently reported [30], particularly when considering that Table IV reports initial proof-of-principle results and network optimization typically follows after demonstrations of feasibility.

Table IV also demonstrates that the DNN that accepts unfocused data achieves consistently higher DSC and contrast when compared with DAS + NLM + BT. The DNN that accepts focused data consistently achieves similar or better DSC results when compared with the state of the art (i.e., DAS + U-Net) and consistently improves image quality (i.e., contrast, gCNR, and PSNR) when compared with DAS + NLM, DAS + BT, DAS + NLM + BT, and DAS + U-Net. These improvements were achieved in parallel rather than sequentially, due to our task-specific training on enhanced B-mode images for simultaneous detection, visualization, and segmentation of anechoic cysts.

## IV. Discussion

The results presented in this article describe our initial successes and challenges with using deep learning to provide useful information directly from a single plane wave insonification. Overall, the proposed task-specific DNN approach is feasible. It is remarkable that acceptable images were achieved prior to the application of receive time delays to compensate for time-of-arrival differences. In particular, the contrast and gCNR of anechoic regions were improved with DNN images over DAS B-mode images created with a single plane wave, tissue SNR was either improved or similar depending on the inclusion of receive delays with subaperture beamforming, and DSC values were similar, regardless of the presence of receive delays. Therefore, the benefits of this approach are that we can train exclusively on simulations of single plane wave transmissions, successfully transfer the trained networks to experimental single plane wave ultrasound data, and produce B-mode images of anechoic targets with superior contrast and gCNR (i.e., two metrics representing improved image quality) and either similar or smoother tissue texture compared with DAS beamforming. An additional benefit is that these image quality improvements were achieved while concurrently extracting segmentation information directly from the raw ultrasound channel data, resulting in similar or better segmentation performance with focused input data when compared with the current state of the art (see Table IV).

Typically, image formation is followed by segmentation, and this sequential process for singular plane wave transmissions generally has the limitations of reduced throughput, as well as poor image quality (which generally produces poor image segmentations). Increasing the number of plane wave transmissions further reduces throughput yet improves image quality at the expense of frame rates. In addition to parallelizing image formation and segmentation, the proposed DNNs offer real-time feasibility (with frame rates of 287–395 Hz based on our hardware and network parameters) as well as improved image quality with a single plane wave transmission. There is additional room for improvement by optimizing the proposed implementation to increase real-time frame rates [30] and to increase *in vivo* segmentation accuracy by including more features during training, which will be the focus of future work.

There are four key observations and insights based on the presented results of applying DNNs to the challenging task of reconstructing sufficient quality images from single plane wave channel data acquisitions. First, we successfully achieved one of the primary goals of our network training, which was to only display structures of interest and otherwise ignore (or deemphasize) surrounding structures. For example, the higher SNR and smoother tissue texture with the unfocused input data align with our goal of deemphasizing unimportant structures for robotic automation. It is additionally advantageous that this network produced images with smoother tissue texture without relying on computationally expensive methods, such as NLM [55] or anisotropic diffusion [56], to generate training data. If speckle is truly desired, we previously demonstrated that a GAN, rather than the FCNN employed in this article, has the potential to produce speckle and provide simultaneous DNN images and segmentation maps from a single input of unfocused plane wave channel data [26].

Similar to the FCNN deemphasis of speckle, the −6-dB cyst in Fig. 6 is poorly visualized in the DNN image. Although the network was trained with anechoic cysts and was not trained to detect hypoechoic cysts, this result suggests that the decoder for the DNN image is somewhat sensitive to echogenicity. However, the hypoechoic cyst in Fig. 6 does not appear in the DNN segmentation output, which suggests that the decoder for the segmentation is selective to the detection of anechoic regions in the input data. Similar task-specific DNN approaches may be devised and implemented to emphasize (as demonstrated with anechoic regions) or deemphasize (as demonstrated with speckle and the low-contrast cyst) other structures of interest for ultrasound-based interventions (e.g., needle tips).

The second insight is that the results of the attenuation study (see Fig. 7) indicate that the DNN trained without simulated depth-dependent attenuation learns to be sensitive to the amplitude of received echoes in order to determine whether a given region is cyst or tissue. However, tissue attenuation confounds this particular network and causes performance deeper into the tissue to drop, as the network confuses the decrease in echo intensity due to tissue attenuation with a decrease in echo intensity due to an anechoic cyst. Counterintuitively, we noticed that performance rises when unrealistic data in the form of the data set without attenuation (in addition to data containing attenuation) is included in the training data set provided to the network. This rise in performance highlights the importance of diversity in the data set—more diverse data yields better generalization. It also showcases that the network has the potential to automatically learn what is useful (e.g., the location-dependent spatial response of the cysts) and discard what is not useful (e.g., the unrealistic lack of attenuation) with additional training data.

Third, although the DNNs were trained with circular, anechoic, cyst-like structures, there was some ability to generally distinguish tissue from cyst in the presence of irregular boundaries (see Fig. 9), although the boundaries themselves seemed to be estimated by the DNN as smooth and circular like the training data. The DNNs also generalized reasonably well to cyst sizes that were not included during training. The network that accepted focused data was additionally able to generalize to data acquired with focused rather than plane wave transmissions, as shown in Fig. 10. There were also generalizations across transmit frequencies and other parameters that differ when comparing the Alpinion L3-8 and L8-17 ultrasound transducer parameters in Table II. In addition, although the DNN that accepts focused data was trained with data containing mostly homogeneous tissue, it was able to generalize to the heterogeneities of the majority of breast tissue surrounding Cysts #1 and #2. One possible reason for poorer performance with Cyst #1 is the presence of bright reflectors in the channel data, which were not included during training. Future work will include additional modeling of heterogeneous tissue. Nonetheless, the observed generalizations are promising for translation to other organs of interest for the proposed DNN (e.g., kidney calyces and ovarian follicles), as well as to other anatomical structures with similar characteristics.

The fourth observation is that the <2.5% mean CV values reported in Section III-B indicate stability and robustness when there is minimal to no perturbations in the input over time. This minimal CV also demonstrates that similar results were produced over the acquisition sequences. Stability and robustness are desirable properties of DNNs [57], which are particularly necessary for biomedical imaging tasks, as imperceptibly small perturbations to the input can often significantly alter the output.

Aside from the common limitations of pilot testing (including few *in vivo* test cases and questions about generalizability to other cases), one limitation observed from the presented results is that smaller cysts presented a greater challenge than larger cysts. This observation is based on the worse DSC, contrast, and gCNR with smaller cysts compared with larger cysts in Fig. 5 and the lower cyst detection ratio for smaller cysts compared with larger cysts in Table III. It is known that the DSC penalizes errors obtained with smaller cysts more severely than errors obtained with larger cysts [58]. While the lower DSCs with smaller cysts are consistent with DSCs achieved with other segmentation approaches [1], [2], the degraded contrast and gCNR with decreased cyst size might be linked to the context–detail tradeoff inherent to deep learning. Prior work [59] demonstrated that CNNs rely on sufficient context to make successful predictions. Linearly interpolating the data to a reduced grid size of 256 × 128 pixels provides each neuron in the CNN with greater context as each neuron sees more of the neighborhood of a particular pixel to make a prediction. However, downsampled data have reduced detail, with the same 2-mm cyst now occupying fewer input pixels in the input to a given neuron. We hypothesize that linearly downsampling to a larger grid size is one possible solution toward addressing the poorer performance with smaller cysts.

The success of the presented results has implications for providing multiple (i.e., more than two) DNN outputs from a single network input. For example, in addition to beamforming and segmentation, deep learning ultrasound image formation tasks have also been proposed for sound speed estimation [60], speckle reduction [43], reverberation noise suppression [61], and minimum-variance directionless response beamforming [62], as well as to create ultrasound elastography images [63], CT-like ultrasound images [64], B-mode images from echogenicity maps [65], and ultrasound images from 3-D spatial locations [66]. We envisage the future use of parallel networks that output any number of these or other mappings to provide a one-step approach to obtain multimodal information, each originating from a singular input of raw ultrasound data.

One example of a specific future application possibility from this perspective, which is also supported by the results presented in this article, is high-frame-rate decision support without requiring multiple different transmit sequences to obtain multiple different output images. More specifically, the parallel B-mode and segmentation information can possibly be extended to include parallel B-mode, segmentation, elastography, sound speed estimation, and CT-like ultrasound images. One could also envision periodically interspersing the more accurate focused DNN results (compared in Fig. 8) among the faster unfocused results to increase the confidence of system performance. These possibilities open new avenues of research to explore the benefits of producing multiple outputs from a single input for parallel clinical, automated, and semiautomated decision making.

## V. Conclusion

This article demonstrates a possible use of DNNs to create ultrasound images and cyst segmentation results directly from raw single plane wave channel data. This approach is a promising alternative to traditional DAS beamforming followed by segmentation. A novel DNN architecture was developed and trained with Field II simulated data containing anechoic cysts insonified by single plane waves. The feature representations learned by the DNN from simulated data were successfully transferred to real phantom and *in vivo* data. This success has future implications for task-specific ultrasound-based approaches to emphasize or deemphasize structures of interest and for producing more than two output image types from a single input image of raw IQ channel data, opening up new possibilities for ultrasound-based clinical, interventional, automated, and semiautomated decision making.

## Figures and Tables

**TABLE I:** Simulated Cyst Image Data Parameters

Parameter	Range	Increment
Radius (*r*)	2-8 mm	1-2 mm
Speed of Sound (*c*)	1420-1600 m/s	10 m/s
Lateral position of cyst center (*x*)	−16 mm - 0 mm	2 mm
Axial position of cyst center (*z*)	40-70 mm	2.5 mm

**TABLE II:** Transducer Parameters

Parameter	Simulated	L3-8	L8-17
Number of Elements	128	128	128
Pitch	0.30 mm	0.30 mm	0.20 mm
Element Width	0.24 mm	0.24 mm	0.11 mm
Kerf	0.06 mm	0.06 mm	0.09 mm
Aperture	38.4 mm	38.4 mm	25.6 mm
Elevational Width	7 mm	7 mm	4 mm
Elevational Focus	35 mm	35 mm	20 mm
Transmit Frequency	4 MHz	4 MHz	12 MHz
Sampling Frequency	100 MHz	40 MHz	40 MHz
Pulse length (cycles)	4	4	1
Center Frequency	5.5 MHz	5.5 MHz	12.5 MHz
Fractional Bandwidth	0.65	0.65	0.65

**TABLE III:** Detection Rate of Simulated Test Set After Training With the Baseline Parameters Listed in Section II-D and Implementing the Exclusion Criteria Listed in Section II-K

Cyst Radius	Total # of Images	# of Images Included	Detection Rate
2 mm	904	624	69%
3 mm	880	880	100%
4 mm	972	972	100%
6 mm	902	902	100%
8 mm	896	896	100%

**TABLE IV:** Performance Comparisons of DAS Beamforming, NLM Speckle Reduction, Binary Thresholding Segmentation Followed by Morphological Filtering (abbreviated as BT), U-Net Segmentation, and DNN Results With Focused and Unfocused Input Data. Processing Times for NLM and BT Were Calculated on a CPU With Remaining Processing Times Calculated on GPUs

		Traditional Sequential Approaches				Proposed DNN Approaches	
	DAS	DAS+NLM	DAS+BT	DAS+NLM+BT	DAS+U-Net	Unfocused DNN Input, *I_d_*	Focused DNN Input, *I_fds_*
Processing Time	0.25 ms	13.29 ms	2.20 ms	15.41 ms	1.72 ms	2.53 ms	3.48 ms
Frame Rate	4,000 Hz	75 Hz	455 Hz	64 Hz	583 Hz	395 Hz	287 Hz
**Phantom**							
DSC	N/A	N/A	0.68 ± 0.09	0.77 ± 0.08	0.92 ± 0.02	0.92 ± 0.03	**0.93 ± 0.01**
Contrast (dB)	−17.14 ± 4.51	−16.08 ± 4.51	−17.14 ± 4.51	−16.08 ± 4.51	−17.14 ± 4.51	**−39.13 ± 5.86**	−37.30 ± 6.86
SNR	1.97 ± 0.22	5.76 ± 2.03	1.97 ± 0.22	**5.76 ± 2.03**	1.97 ± 0.22	4.96 ± 1.84	1.82 ± 0.37
gCNR	0.77 ± 0.07	0.94 ± 0.03	0.77 ± 0.07	0.94 ± 0.03	0.77 ± 0.07	0.93 ± 0.08	**0.95 ± 0.03**
PSNR (dB)	N/A	17.22 ± 0.99	N/A	17.22 ± 0.99	N/A	19.33 ± 0.83	**23.07 ± 0.86**
***In Vivo* Cyst #1**							
DSC	N/A	N/A	0.68 ± 0.00	0.76 ± 0.00	**0.83 ± 0.01**	0.77 ± 0.07	0.82 ± 0.03
Contrast (dB)	−13.61 ± 2.36	−11.43 ± 2.48	−13.61 ± 2.36	−11.43 ± 2.48	−13.61 ± 2.36	**−25.72 ± 9.25**	−25.30 ± 3.69
SNR	1.27 ± 0.07	1.76 ± 0.15	1.27 ± 0.07	1.76 ± 0.15	1.27 ± 0.07	**3.94 ± 0.59**	1.12 ± 0.21
gCNR	0.56 ± 0.03	0.76 ± 0.03	0.56 ± 0.03	0.76 ± 0.03	0.56 ± 0.03	0.75 ± 0.14	**0.89 ± 0.04**
PSNR (dB)	N/A	16.47 ± 0.01	N/A	16.47 ± 0.01	N/A	15.05 ± 0.86	**18.86 ± 0.31**
***In Vivo* Cyst #2**							
DSC	N/A	N/A	0.78 ± 0.01	**0.81 ± 0.00**	0.72 ± 0.07	-	0.79 ± 0.02
Contrast (dB)	−18.27 ± 2.59	−16.40 ± 2.55	−18.27 ± 2.59	−16.40 ± 2.55	−18.27 ± 2.59	-	**−31.62 ± 2.56**
SNR	1.29 ± 0.13	3.08 ± 0.38	1.29 ± 0.13	**3.08 ± 0.38**	1.29 ± 0.13	-	1.39 ± 0.12
gCNR	0.75 ± 0.09	0.94 ± 0.02	0.75 ± 0.09	0.94 ± 0.02	0.75 ± 0.09	-	**0.96 ± 0.01**
PSNR (dB)	N/A	19.45 ± 0.02	N/A	19.45 ± 0.02	N/A	-	**19.58 ± 0.16**
